# Beneficial effects of reduced soft tissue vibrations with compression garments on delayed neuromuscular impairments induced by an exhaustive downhill run

**DOI:** 10.3389/fspor.2025.1516617

**Published:** 2025-05-07

**Authors:** Robin Gassier, Loïc Espeit, Antoine Ravel, Pierre Beaudou, Robin Trama, Pascal Edouard, Arsène Thouze, Léonard Féasson, Frédérique Hintzy, Jérémy Rossi, Christophe Hautier

**Affiliations:** ^1^ Inter-University Laboratory of Human Movement Biology, Universite Claude Bernard Lyon 1, LIBM, Villeurbanne, France; ^2^ Inter-University Laboratory of Human Movement Biology, Université Jean Monnet Saint-Etienne, Saint-Etienne, France; ^3^ Human Performance Laboratory, Faculty of Kinesiology, University of Calgary, Calgary, AB, Canada; ^4^ Department of Clinical and Exercise Physiology, Sports Medicine Unit and Myology Unit, University Hospital of Saint-Etienne, Faculty of Medicine, Saint-Etienne, France; ^5^ Department of Movement Sciences, Décathlon, Lille, France; ^6^ Inter-University Laboratory of Human Movement Biology, University Savoy Mont-Blanc EA7424, Le Bourget du lac, France

**Keywords:** acceleration, fatigue, muscle damage, trail running, wavelet

## Abstract

**Introduction:**

Soft tissue vibrations (STV) have been extensively researched for their effects on muscle fatigue and damage, but their influence during running remains unclear. As compression garments are known to lower STV, they have shown benefits on acute neuromuscular responses to downhill running. However, an in-depth analysis of changes in STV has never been proposed, and previous protocols did not overcome the repeated bout effect. This study aims to investigate whether compression shorts could reduce STV parameters and related neuromuscular impairments using a unilateral compression protocol.

**Materials and methods:**

Twenty healthy men performed a downhill run until exhaustion while wearing shorts that compressed one thigh with the contralateral leg serving as a control. Foot–ground impacts (FGI), STV, and muscle activation of the *Vastus Lateralis* were measured on both legs while running using accelerometers to obtain FGI and STV, and surface electromyographic sensors (EMG) for muscle activation. Time–frequency analyses were applied to acceleration and EMG signals with statistical non-parametric mapping applied to the continuous data to assess time and compression effects. Neuromuscular parameters such as maximal voluntary contraction torque, voluntary activation, and torque evoked by 10 and 100 Hz doublet stimulation were assessed before, after, and 48 h post exercise, alongside perceived fatigue and muscle soreness. Mixed linear models and paired Student's t-tests were used to analyze neuromuscular outcomes.

**Results:**

While results showed that both FGI and STV magnitude increased during the run by 19.7% (*P* = 0.002) and 17.8% (*P* = 0.003), respectively, compression reduced the magnitude and frequency of STV by 15.1% (*P* = 0.013) and 11.7% (*P* = 0.001), respectively, without influencing FGI or muscle activation. Although neuromuscular parameters were altered in both legs, losses of torque evoked by 10 and 100 Hz doublets were lower in the compressed leg 48 h post exercise (*P* < 0.001 and *P* = 0.001, respectively).

**Conclusion:**

This study revealed the potential of compression garments to act as a mechanical support that attenuates high-frequency STV during downhill running and mitigates subsequent delayed neuromuscular alterations.

## 1 Introduction

Running is one of the most widely practiced sports, attracting hundreds of millions of enthusiasts because of its positive effects (1). The repetitive nature of running leads to numerous foot–ground impacts (FGI), generating shockwaves that propagate through the bones and soft tissues, including muscles, tendons, adipose tissue, ligaments, and skin. These shockwaves cause temporary deformation of the soft tissues, resulting in oscillations as the tissues return to their resting position. These oscillations, known as soft tissue vibrations (STV), exhibit viscoelastic behavior and are characterized by specific frequencies, amplitudes, and damping properties (2).

While the understanding of the effects of repetitive transient vibrations is limited, exposure to continuous vibrations of high amplitude and/or frequency has been shown to increase muscle activity (3), elevate oxygen consumption (4), accelerate muscle fatigue, and induce muscle damage (5). Specifically, muscle fatigue is defined as any exercise-induced reduction in the ability of a muscle to generate force or power (6). It is often assessed using the electrical peripheral nerve stimulation protocol described by Millet et al. (7), which quantifies the loss of muscle contractility and voluntary activation, typically accompanied by a measurement of the perceived fatigue (8). In addition, muscle damage is further evidenced by prolonged decreases in both voluntary and electrically stimulated force, along with the onset of muscle soreness (9).

Given the potential impact of STV on muscle fatigue and damage, several studies have investigated the effect of sports equipment on reducing these vibrations. Authors have examined how footwear can reduce STV, but evidence of a link with neuromuscular fatigue remains inconclusive (10–12). In addition, compression garments (CG) have been proposed as a method to reduce STV during dynamic activities (13–15). Although some studies suggested that CG could reduce muscle fatigue and damage, they did not directly measure the effect of CG on STV (16–18). Only Ehrström et al. (19) have simultaneously measured STV, the associated muscle fatigue and damage, with and without CG. Downhill running was selected as it amplifies STV (19). The authors reported that wearing high-pressure CG during 40 min of downhill running resulted in fewer neuromuscular alterations in knee extensors, both immediately and 1 day post exercise, in well-trained trail runners. However, their analysis of STV was limited to the time domain, whereas more advanced time–frequency analyses, such as wavelet transforms, can provide deeper insights into the frequency content and damping characteristics of STV (20–22). By introducing wavelet transform and statistical parametric mapping analysis, this study intended to provide a novel and more comprehensive characterization of shock-induced STV during downhill running and the effect of CG, bridging the limitations of time domain analysis.

Moreover, the design of Ehrström et al.'s study did not fully account for the repeated bout effect, which may result in an under- or overestimation of the neuromuscular alterations observed (23). To address this limitation and reinforce previous observations, it seems appropriate to use an experimental design that involves a single downhill running bout with one thigh compressed using CG and the other thigh uncompressed serving as a control (18).

In light of the gaps in current understanding, this study aims to investigate whether wearing CG during an exhaustive downhill run could minimize STV of the *Vastus Lateralis*, and consequently reduce muscle fatigue and muscle damage in the knee extensors. In addition, FGI were measured using accelerometers to confirm that unilateral CG use was not associated with changes in impact forces. We hypothesized that reducing STV by wearing CG during a downhill run would lead to less muscle fatigue and muscle damage.

## 2 Materials and methods

### 2.1 Overall study design

**Figure 1:**
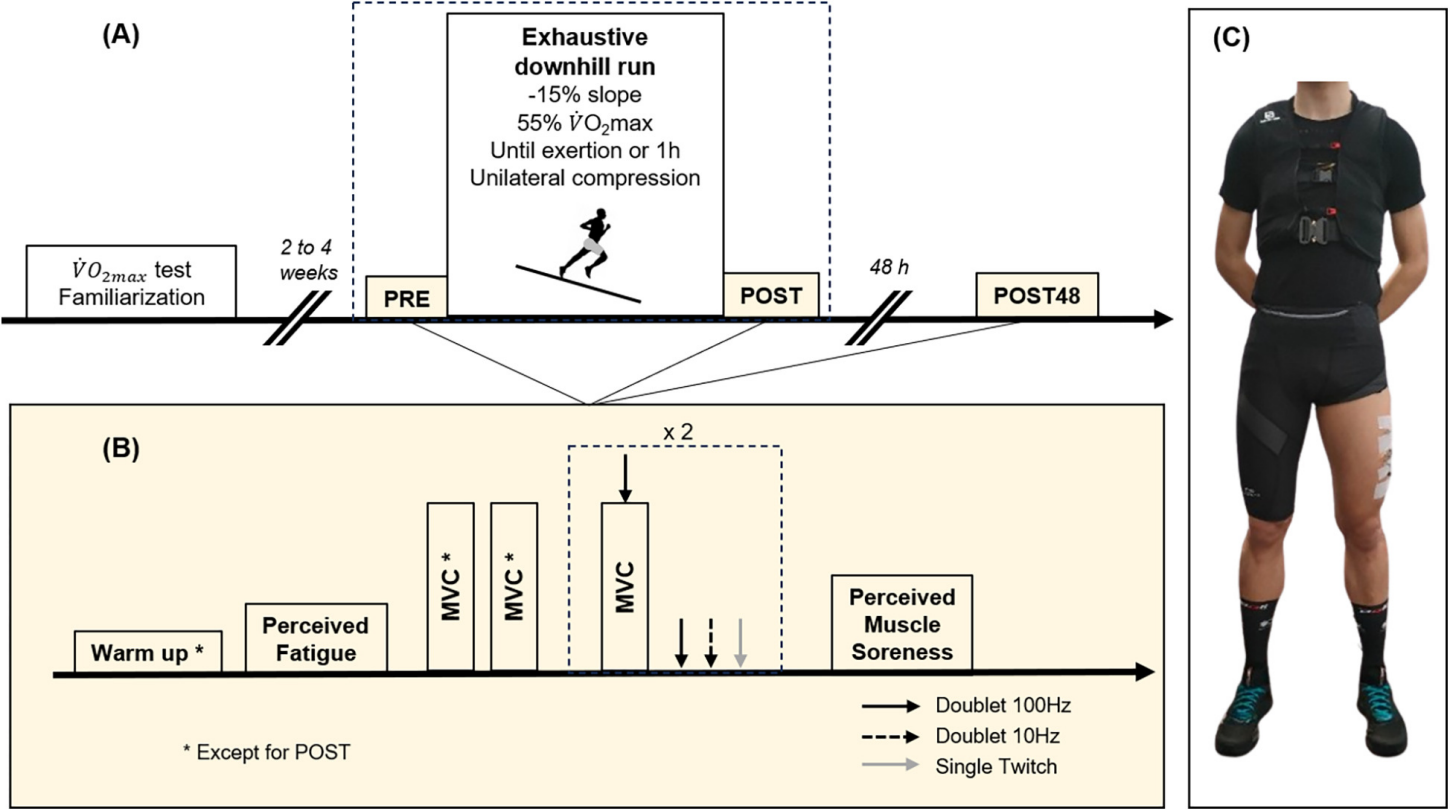
**(A)** Overall protocol and **(B)** neuromuscular testing, perceived fatigue and muscle soreness assessment. **(C)** Overview of how the participants were equipped. MVC, maximal voluntary contraction.

The experiment took place over three sessions: (1) a familiarization to neuromuscular testing and maximal oxygen consumption $\left(\dot{V}O_{2\max}\;\right)$) assessment to establish the velocity of downhill running (VDR); (2) an exhaustive downhill run with unilateral thigh compression during which STV and muscle activation measurements were completed. It was preceded and followed by neuromuscular testing, perceived fatigue, and perceived muscle soreness assessment; (3) finally, a neuromuscular testing, perceived fatigue, and perceived muscle soreness assessment 48  h later. An overview is given in Figure 1.

### 2.2 Participants

A total of 20 active men volunteered to participate in this study (Table 1). All participants gave written informed consent in accordance with the Declaration of Helsinki. The protocol was approved by the French Ethical Research committee (Comité de Protection des Personnes no. 2021-A01453-38). Participants had no injuries in the 6 months before the experiment and practiced flat running at least once a week; those who participated in uphill or downhill running were not included in this study. The sample size was calculated using G*Power software G*Power (RRID: SCR_013726), based on the study by Ehrström et al. (19), who obtained an effect size (ES) of 0.86 for the effectiveness of limiting the increase in *Vastus Lateralis* vibration amplitude, or an effect size of 0.83 for the efficacy of CG in limiting the onset of central fatigue. Based on a two-factor analysis of variance for two groups and three measurement times, with a risk *α* = 0.05 (adjusted using a Bonferroni correction for five variables) and a statistical power of 80%, the recommended sample size was 16 healthy volunteers. To ensure complete data on 16 participants, four more volunteers were recruited, allowing a 20% dropout rate (considering signal noise, unforeseen events, participants not returning on the second day), while maintaining statistical power.

**Table 1:** Participants characteristics.

Variable	Mean ± standard deviation
Age (years)	24.4 ± 5.6
Height (cm)	178.7 ± 6.3
Body mass (kg)	72.2 ± 6.4
Exercise (h/week)	6.0 ± 3.6
Running mileage (km/week)	15.8 ± 9.5
$\dot{V}O_{2\max}$ (ml/kg/min)	58.4 ± 6.1
Maximal aerobic speed (km/h)	18.2 ± 1.5
Body fat (%)	12.8 ± 3.4
Thigh girth (cm)	47.8 ± 4.4
Thigh skinfold (mm)	7.5 ± 3.3

### 2.3 Experimental procedures

#### 2.3.1 Familiarization and $\dot{V}O_{2\max}$ assessment

The familiarization and $\dot{V}O_{2\max}$ assessment session started with a 2 min warm-up walking at 4 km/h on a motorized treadmill (Gymrol S2500 HEF Techmachine Andrézieux Bouthéon), followed by a 6 min run at a comfortable speed (*v*) to measure the energy cost of level running (*Cr*_0_) using a gas analyzer (Metamax 3B_R2, Cortex Biophysik, Leipzig, Germany) worn throughout the protocol Equation (1). This was followed by a 4-min recovery walk at 4 km/h, before moving on to the $\dot{V}O_{2\max}$ test (increment of 0.5 km/h each 30 s starting at 6 km/h). $\dot{V}O_{2\;\max}$ was determined from the highest mean value over a 15 s interval.

(1) $$\begin{array}{c} Cr_{0}=\frac{\dot{V}O_{2}\;}{v} \end{array}$$

Establishing *Cr*_0_ made it possible to define the downhill energy cost of running (*Cr_i_*) based on Equation (2) (24), where *i* is the incline of the terrain (%).

(2) $$\begin{array}{c} Cr_{i}=Cr_{0}+7.44i^{5}-1.45i^{4}-2.07i^{3}+2.22i^{2}+0.93i \end{array}$$

Finally, the individual VDR was determined for a run at 55% $\;\dot{V}O_{2\max}$ on a −15% slope Equation (3).

(3) $$\begin{array}{c} \mathrm{VDR}=0.55\times \frac{\dot{V}O_{2\max}}{Cr_{\left(-15\right)}} \end{array}$$

For example, a participant ran for 6 min at a comfortable speed of v=7.5km/h=125m/min with a measured $\dot{V}O_{2}=$ $29\;\mathrm{ml}\;O_{2}/\min/\mathrm{kg}\;$*,* and a measured $\dot{V}O_{2\max}=62\;\mathrm{ml}\;O_{2}\;/\min/\mathrm{kg}$ at the end of the incremental test. His VDR was calculated using Equations 4–6:

(4) $$\begin{array}{c} Cr_{0}=\frac{\dot{V}O_{2}\;}{v}=\;\frac{29}{125}=0.232\;\mathrm{ml}\;O_{2}/(\mathrm{kg}\cdot m) \end{array}$$

(5) $$\begin{array}{rl} Cr_{-0.15} & =0.232+7.44\times {\left(-0.15\right)}^{5}-1.45\times {\left(-0.15\right)}^{4}\\ & -2.07\times {\left(-0.15\right)}^{3}+2.22\times {\left(-0.15\right)}^{2}+0.93\times (-0.15)\; \end{array}$$

$$\begin{array}{c} Cr_{-0.15}=0.148\;\mathrm{ml}\;O_{2}/(\mathrm{kg}\cdot m) \end{array}$$

(6) $$\begin{array}{c} \mathrm{VDR}=0.55\times \frac{62}{0.148} \end{array}$$

VDR=230.41m/min=13.8km/h

During this first session, participants also became familiar with the neuromuscular testing, which included maximal voluntary contractions (MVCs) of the knee extensors with and without femoral nerve electrical stimulation. Participants were also asked not to change their sleeping, eating, or exercise habits in the 48 h preceding the experiment.

#### 2.3.2 Exhaustive downhill run session

During the exhaustive downhill run session, participants underwent prerun tests (PRE), then a downhill run to exhaustion, immediately followed by postrun tests (POST). They performed a standardized warm-up of 5 min at self-paced submaximal intensity on a cycle ergometer. After this, PRE consisted of neuromuscular testing of the knee extensors of both legs in a randomized order, perceived muscle soreness, and perceived fatigue questionnaires. Volunteers were fitted with surface electromyographic sensors (EMG) each *Vastus Lateralis* (Delsys TrignoTM Wireless EMG System quattro sensor, bipolar Ag: AgCI surface, 2 cm interelectrode distance, Delsys, Inc., Boston, MA, USA) according to the SENIAM guidelines (sampling rate: 2222 Hz, rejection ratio >80 dB). Four triaxial accelerometers (3273A4 Series, Dytran Instrument, Marilla St, Chatsworth, CA, USA; mass 3 g; sampling frequency 2222 Hz; range ± 100 g; sensitivity 50 mV/g) were taped (*y*-axis oriented vertically) to the muscle bellies of both *Vasti Lateralis* to record the STV and to the heel cups of standardized shoes (Arc'teryx Norvan LD, hardness: Asker 40C) to record the FGI (25).

Participants wore CG (Decathlon garments, Villeneuve d'Ascq, France) on one randomly selected thigh (compressed situation: COMP), and the other was the control one (CONT) with randomization conducted by the survey team using a counterbalanced computer-generated method. The level of compression was measured (Inflatable pressure sensor, Chattanooga Group Inc., Hixson, TN, USA) at 15.9 ± 2.3 mmHg.

Once equipped, participants performed two maximal squat jumps to normalize EMG signals (26). They were asked to pause in a low position at 90° knee flexion; arms remained across their chest throughout the movement and a recovery time of between 2 and 3 min was provided between jumps. Afterward, they ran for 3 min at −15% at their own pace, to familiarize with downhill running on treadmill (Venus 200/100r, HP cosmos, Germany) before starting the exhaustive downhill run. Participants ran at VDR to exhaustion or for 1 h if the effort could be maintained until then (only three participants reached the 1-h limit). The slope, duration, and speed of the downhill run were established with regard to previous studies (19) and observations made by Bontemps et al. (27) concerning their effects on neuromuscular alterations. Accelerometric and EMG data were collected for 30 s every 3 min. All signals were recorded and synchronized with Qualisys Track Manager—Software 2022.2 (Qualisys, Göteborg, Sweden). They were detected and analyzed with customized scripts in Matlab (R2022b, The Mathworks, Natick, MA, USA; MATLAB, RRID:SCR_001622). The data collected during the second recording of 30 s [i.e., 6 min after the beginning of the race to ensure stabilization of biomechanical parameters (10)] were considered “FRESH” state data, while the data of the last recording before exhaustion were considered “FATIGUE” state of the participants.

Within 3.8 ± 1.1 min of finishing the exhaustive downhill run, the participants performed the POST evaluation in the same order as PRE.

#### 2.3.3 Post 48 h assessments

In line with previous recommendation and observations (9, 27), the neuromuscular testing (POST-48) was conducted 48 h later, in the same order as PRE and POST. Perceived muscle soreness and fatigue were monitored daily after the exhaustive downhill run for 7 days. Participants were instructed not to perform any recovery interventions such as massage, icing, or nutritional supplements.

#### 2.3.4 Neuromuscular testing

The protocol described by Millet et al. (7) was used for neuromuscular testing of the knee extensors. It has subsequently been used on various occasions to quantify neuromuscular fatigue (7, 19).

##### 2.3.4.1 Installation of participants

Participants seated on an isometric knee dynamometer (ARS dynamometry, SP2, Ltd., Ljubljana, Slovenia) with hips and knees at 90° of flexion. The assessed leg was secured to the force transducer with a non-compliant strap just above the intermalleolar axis, while the pelvis was also securely restrained to the ergometer ensuring participants' stability during the assessment. Surface EMG signals of the *Vastus Lateralis* muscle were recorded using 35 mm diameter pairs of self-adhesive electrodes (McKesson Medical-Surgical Inc., Richmond, VA, USA) in bipolar configuration with 35 mm interelectrode distance, placed according to SENIAM recommendations (28) and the reference electrode attached to the patella. Electrical femoral nerve stimulation was achieved using a self-adhesive 35 mm diameter surface cathode manually pressed into the femoral triangle (McKesson Medical-Surgical Inc., Richmond, VA, USA) and a 50–100 mm self-adhesive stimulation electrode (Compex, Enovis Global, Ecublens, Switzerland) located in the gluteal fold. Placement of all the electrodes was marked to ensure a reliable placement on PRE, POST, and POST-48 tests.

##### 2.3.4.2 Stimulation intensity

Single square-wave electrical stimuli of 1 ms duration and 400 V maximal voltage were delivered via a constant current stimulator (DS7R Digitimer, Welwyn Garden City, Hertfordshire, United Kingdom). The optimal stimulation intensity was found, by progressively stimulating the femoral nerve until reaching maximal evoked torque and maximal M-wave. It was reassessed before each session, and 130% of this optimal intensity was used for femoral nerve stimulation during the neuromuscular testing.

##### 2.3.4.3 Neuromuscular testing protocol

The neuromuscular testing was conducted after a warm-up of 10 submaximal isometric contractions of the knee extensors for PRE and POST-48 sessions. The protocol consisted of isometric MVC. It was followed 1 min later by an MVC with a superimposed doublet at 100 Hz (Db100_sup_) by peripheral nerve stimulation delivered on an isometric plateau (identified with real-time visual feedback). Then, on the relaxed muscle, 3 s after the end of the MVC and every 3 s, a high-frequency doublet at 100 Hz (Db100) and a low-frequency doublet at 10 Hz (Db10) were induced. Participants were asked to fold their arms over their chest and were strongly encouraged during the MVCs and then asked to relax during the potentiated electrical stimulation doublet. The entire set was repeated twice for each leg. EMG data were recorded using a PowerLab system (16/30-ML880/P; ADInstruments, Bella Vista, NSW, Australia) with a sampling frequency of 2000 Hz. The EMG signal was amplified with an octal bioamplifier (Octal Bioamp, ML138; ADInstruments) with a bandwidth frequency ranging from 5 to 500 Hz (common mode rejection ratio, 85 dB; gain, 500), transmitted to the computer, and analyzed with LabChart 8 software (ADInstruments, RRID:SCR_023643).

#### 2.3.5 Perceived muscle soreness and fatigue

Perceived muscle soreness was assessed using a visual analog scale consisting of a 10 cm continuous line going from 0 “no pain” to 10 “very, very painful” (29). Participants were asked to record the severity of muscle soreness of the anterior thigh of both legs in three different positions: in resting position while seated, in a position laying prone and actively flexing the knee aiming to stretch the knee extensors, and during a full squat.

The participants' perceived fatigue was also collected on a 10 cm visual analog scale. They reported their global fatigue from 0 “no fatigue” to 10 “exhausted” (7).

### 2.4 Data analysis

#### 2.4.1 Neuromuscular testing data

MVC torque was defined as the maximal torque produced from two trials. Db10 and Db100 evoked torques were retained for analysis from the trial with the maximal superimposed twitch torque (Db100_sup_). The ratio of Db10 out of Db100 was calculated (Db10/Db100). The percentage of voluntary activation (%*VA*) was then calculated from Equation (7), where *T*_before_ is the torque just before the superimposed double twitch, and *T*_max_ is the maximum torque attained during the contraction (30).

(7) $$\begin{array}{c} \text{\%}VA=100-\left[(Db100_{\sup}-T_{\mathrm{before}})\times \frac{T_{\mathrm{before}}/T_{\max}}{Db100}\times 100\right] \end{array}$$

Absolute and relative acute neuromuscular effects were established by subtracting POST values from PRE values, while delayed effects were established by subtracting POST-48 values from PRE values (7, 30).

#### 2.4.2 Acceleration data

**Figure 2:**
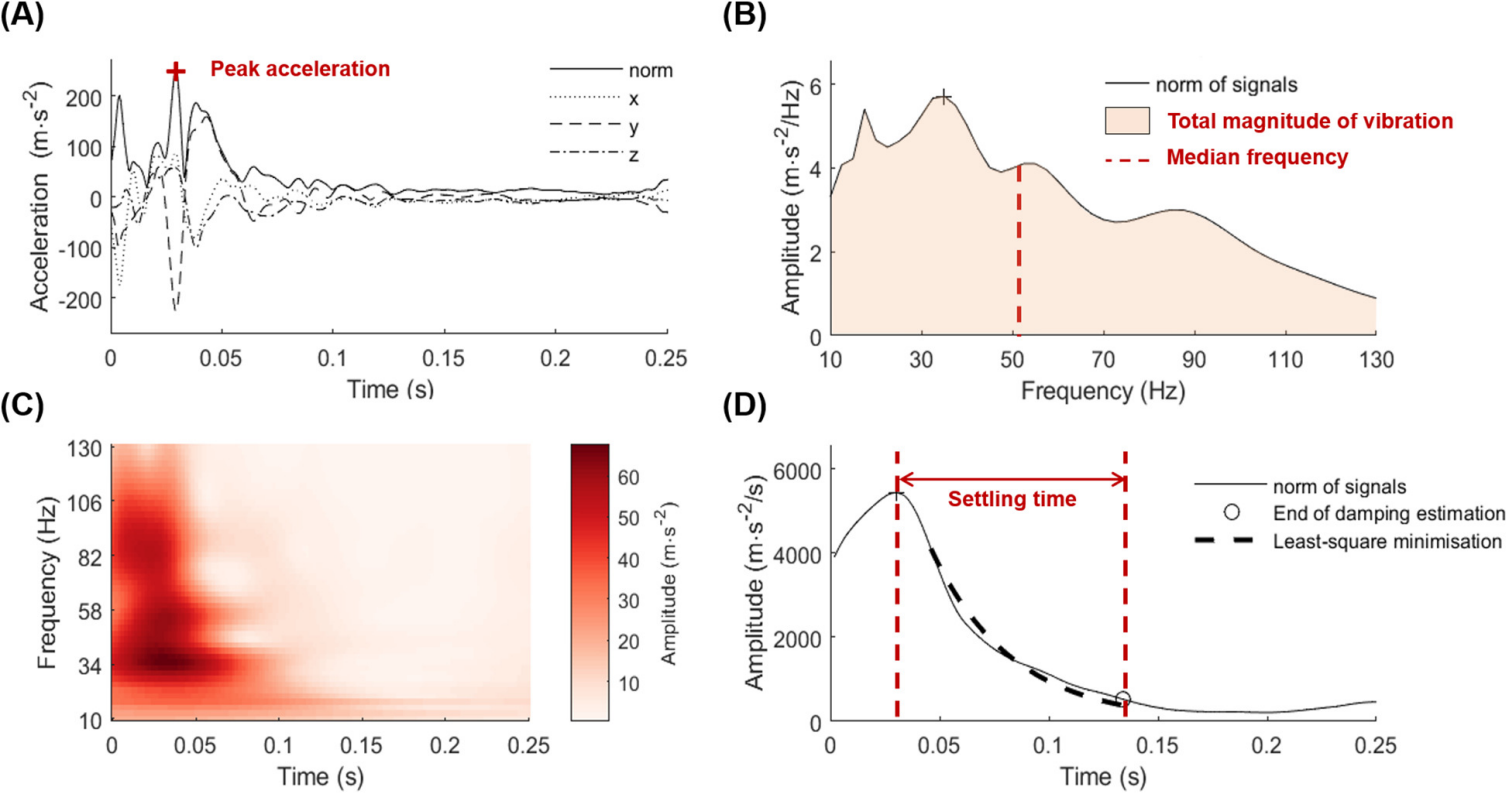
Acceleration data analysis: **(A)** raw components and norm of acceleration signal in the time domain, **(B)** frequency domain spectrum after wavelet transforms, **(C)** coefficients map after wavelet transform analysis, and **(D)** time domain spectrum after wavelet transforms.

The raw acceleration data were filtered using a fourth-order band-pass Butterworth filter at 10–400 Hz to remove the movement artifacts and electronic noise. FGI was detected with a threshold of 90 m · s^−2^ based on the norm of the three axes of the heel accelerometers. Acceleration data from FGI detection up to 250 ms afterward were considered for analysis. The norm of the signal was calculated based on filtered data of the three axes. The maximum of this norm was considered as the peak acceleration (ACC_PEAK_ in m · s^−2^) (Figure 2A).

A time–frequency analysis of the vibration signal was performed using a continuous wavelet transform from a Morse mother wavelet (with a symmetry parameter *γ* equal to 3, and a time bandwidth *P*^2^ equal to 60) (25, 31) (Figure 2C). The wavelet coefficients from this transform represented the magnitude of the vibration at each frequency from 10 to 130 Hz over 250 ms after the impact. A frequency spectrum was computed by integrating the coefficients by the time and the median frequency (ACC_MDF_ in Hz) was quantified as the frequency splitting the spectra in two equal parts (Figure 2B). The total magnitude of the vibration (TMV, in m · s^−2^/Hz/s as it is an acceleration integrated in time and frequency) was calculated with the frequency integral of the spectra (25) (Figure 2B). The settling time (ST in ms) of each transient vibration was calculated as the time required for the signal to decay from the peak acceleration to an amplitude of 10% of that peak (20, 31) (Figure 2D).

#### 2.4.3 EMG data

Data were filtered using a fourth-order band-pass Butterworth filter at 50–450 Hz, and root-mean square (RMS) was calculated on a 100 ms sliding rectangular window. The onset and offset of each EMG burst were detected by a threshold set to be 10% of the local peak of the maximum amplitude (32). The EMG_RMS_ of each burst was then normalized to the peak of EMG_RMS_ of the counter movement jump task. The mean of normalized EMG_RMS_ value was calculated for each burst (EMG_MEAN_), as well as the peak (EMG_MAX_) and the signal integral (iEMG).

A continuous wavelet transform analysis was conducted on EMG signals (Morse, 3,60) by applying the methodology of Zhang et al. (33). Computing the modulus of the coefficient given by each node of the wavelet transform enables the establishment of a time–frequency map. The median frequency (EMG_MDF_ in Hz) was calculated by the integration of the time–frequency map by the time.

### 2.5 Statistics

#### 2.5.1 Statistical non-Parametric Mapping

Statistical non-Parametric Mapping (SnPM) was conducted on the frequency spectra, computed by integrating the coefficients by the time obtained from the wavelet transform. These spectra were analyzed with a two-way repeated ANOVA (time [FRESH vs. FATIGUE] × compression [COMP vs. CONT]), and *post-hoc t*-tests with Bonferroni correction were carried out if the analysis of variance indicated a significant difference. These analyses were performed on vibration frequency spectra for FGI and STV of the *Vastus Lateralis*, producing a total of two variables. The alpha risk was defined as 0.05/2 = 0.025. The fctSnPM package on MATLAB performs statistical inferences (F- or *t*-values) with a non-parametric approach called permutation tests, to recalculate the distribution of each node and determine the significance threshold of the spectrum (34). Consequently, 10000 permutations were conducted to achieve computational stability [at least 100/*α*, e.g., 4000 recommended by Nichols and Holmes (35)].

#### 2.5.2 Mixed linear model

Mixed linear models (36) were used to investigate the effects of compression and time on extracted variables. For each model, participants were considered as random effect because of the dependence among observations (i.e., repeated measures). As participants may also not share the same baseline, and because the fixed effects may differ among participants, the fixed effects were also set as random intercepts and slopes. Assumptions of normality, homoscedasticity, and linearity of the linear-mixed model residuals were graphically controlled and checked with a residual diagnostics tool for hierarchical (multilevel/mixed) regression models (DHARMa). *P*-values were obtained by performing conditional *F*-tests with Satterthwaite's degrees of freedom correction (36). In case of interaction of fixed effects, a Tukey HSD *post-hoc* test with Satterthwaite's degrees of freedom correction was performed on the model to determine the direction of differences. A Bonferroni sequential correction was applied to the *p*-values to avoid false positives.

The first model used two fixed effects [time (FRESH vs. FATIGUE) × compression (COMP vs. CONT)], with participants and fixed effects indicated as a random intercepts and slopes. This analysis was performed on vibration (TMV, ACC_PEAK_, ACC_MDF_, ST for the *Vastus Lateralis* and FGI) and muscle activation (EMG_MEAN_, EMG_MAX_, EMG_MDF_, iEMG) during the downhill run.

The second model used two fixed effects [time (PRE − POST − POST48) × compression (COMP vs. CONT)], with participants and fixed effects indicated as random intercepts and slopes. This analysis was performed on neuromuscular parameters (MVC, %VA, Db10, Db100, and Db10/Db100).

The final model used two fixed effects [time (PRE, POST, DAY1, DAY2, DAY3, DAY4, DAY5, DAY6, DAY7) × compression (COMP vs. CONT)], with participants and fixed effects indicated as random intercepts and slopes. Assumptions of normality, homoscedasticity, and linearity of the linear-mixed model residuals were not respected; therefore, non-parametric Friedman's tests followed by paired Wilcoxon with Bonferroni correction were performed. This analysis was performed on perceived muscle soreness (seated, stretched, squat) and perceived fatigue.

All models were performed using the package lme4 (36) of R software (R 3.5.0, RCore Team, Vienna, Austria, RRID: SCR_001905).

#### 2.5.3 Paired Student and Wilcoxon tests

A Student paired *t*-test was performed to compare the percentage of variation of neuromuscular parameters generated by the exhaustive downhill run between COMP and CONT conditions, and also to identify any difference at baseline. This analysis was performed on neuromuscular parameters (MVC, %VA, Db10, Db100, and Db10/Db100). When normality or homogeneity were n't met, a non-parametric Wilcoxon test was conducted, and Bonferroni correction was applied.

#### 2.5.4 Effect sizes

Effect sizes were calculated between the different comparisons using Cohens' *d* value for repeated measures, with pooled standard deviations and Hedges' g correction for non-normal distributions in SnPM analysis (37).

## 3 Results

All participants completed the entire protocol (*n* = 20). The average of running time and speed was 39.8 ± 13.2 min and 13.0 ± 1.4 km/h, respectively.

### 3.1 Foot ground impacts and soft tissue vibrations during the exhaustive downhill run

**Figure 3:**
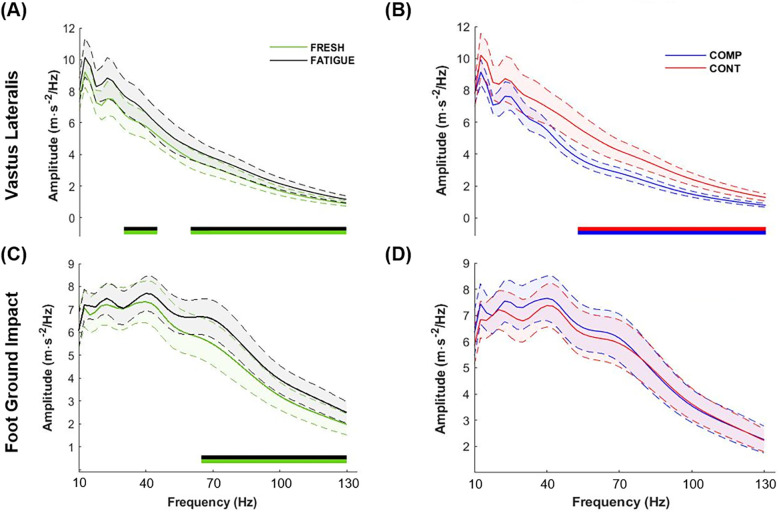
Time (FRESH − FATIGUE) and compression (COMP − CONT) effects on frequency representation (mean ± 95% confidence interval) of foot–ground impact and soft tissue vibrations of the *Vastus Lateralis.* From left to right and from top to bottom: **(A)** Time effect on soft tissue vibrations of the *Vastus Lateralis*, **(B)** compression effect on *Vastus Lateralis* soft tissue vibrations, **(C)** time effect on foot–ground impact, and **(D)** compression effect on foot–ground impact. Below the curves, significant differences between two conditions are represented by the two-colored bands. If one color is above another, this condition generates more energy at these frequencies than the other.

Acceleration analysis was carried out on 16 participants due to data loss caused by the high number and intensity of impacts and vibrations, as well as sweat, which led to movement or loss of contact between the accelerometers and the muscles during the exhaustive downhill run. The SnPM ANOVAs revealed no time–compression interaction. A time effect was observed for both FGI (Figure 3C) and STV of the *Vastus Lateralis* (Figure 3A). The amplitude of the FGI was higher in the end of exhaustive downhill run with a significant difference above 65 Hz. STV's amplitude was also greater from 30 to 45 Hz and above 60 Hz just before exhaustion (Figure 3A). The total magnitude of vibration and median frequency of STV of the *Vastus Lateralis* were significantly increased with time (*P* = 0.003 and *P* = 0.02, respectively), as well as total magnitude and median frequency of FGI (*P* = 0.002 and *P* < 0.001, respectively) (Table 2).

**Table 2:** Magnitude of compression effect and time effect on FGI, STV, and EMG of the *Vastus Lateralis*.

Biomechanical variables in running		Compression effect					Time effect				
		CONT	COMP	%*Δ*	Effect size	*p*-value	FRESH	FATIGUE	%*Δ*	Effect size	*p*-value
STV of VL	TMV (m.s^−2^/Hz/s)	561.0 ± 241.1	425.2 ± 112.9	−15.1 ± 30.6	−0.626	**0.013**	452.8 ± 170.6	533.4 ± 218.9	19.7 ± 26.0	0.371	**0.003**
	ACC_MDF_ (Hz)	44.0 ± 4.9	38.6 ± 4.4	−11.7 ± 10.9	−1.135	**0.001**	40.8 ± 5.4	41.8 ± 5.4	2.9 ± 6.2	0.198	**0.021**
	ACC_PEAK_ (m.s^−2^)	284.1 ± 119.9	240.0 ± 74.5	−4.0 ± 39.1	−0.420	0.127	248.7 ± 97.6	275.4 ± 105.0	15.4 ± 32.1	0.255	0.080
	ST (ms)	0.101 ± 0.020	0.099 ± 0.023	−3.1 ± 35.2	−0.090	0.788	0.100 ± 0.021	0.100 ± 0.022	3.2 ± 27.6	0.006	0.975
FGI	TMV (m.s^−2^/Hz/s)	646.2 ± 227.5	641.3 ± 248.4	0.1 ± 21.8	−0.020	0.861	607.7 ± 251.8	679.8 ± 217.7	17.8 ± 20.1	0.285	**0.002**
	ACC_MDF_ (Hz)	54.0 ± 6.5	53.3 ± 7.1	−1.3 ± 7.5	−0.110	0.405	51.6 ± 7.0	55.7 ± 6.1	8.6 ± 7.6	0.602	<**0.001**
	ACC_PEAK_ (m.s^−2^)	344.2 ± 149.9	339.9 ± 156.0	0.5 ± 23.9	−0.028	0.764	328.2 ± 165.4	355.9 ± 138.1	18.7 ± 30.1	0.170	0.156
	ST (ms)	0.093 ± 0.018	0.094 ± 0.015	3.8 ± 19.3	−0.101	0.624	0.097 ± 0.019	0.090 ± 0.013	5.3 ± 14.4	0.390	0.050
EMG	EMG_MEAN_ (%)	29.6 ± 9.2	29.6 ± 11.9	10.5 ± 53.0	0.004	0.993	28.6 ± 9.9	30.5 ± 11.3	8.6 ± 22.9	0.412	0.222
	EMG_MDF_ (Hz)	160.3 ± 20.7	152.4 ± 16.2	−3.8 ± 12.6	−0.407	0.211	155.0 ± 17.9	157.7 ± 20.0	1.8 ± 6.2	0.130	0.228
	EMG_MAX_ (%)	47.1 ± 14.4	47.5 ± 19.1	11.3 ± 53.4	0.025	0.949	45.3 ± 15.0	49.4 ± 18.4	10.4 ± 21.2	0.228	0.107
	iEMG (%)	7.5 ± 2.5	7.6 ± 2.9	13.4 ± 52.8	0.051	0.881	7.2 ± 2.4	7.9 ± 2.9	11.1 ± 21.8	0.248	0.089

COMP, compressed condition; CONT, control condition; STV, soft tissue vibrations; FGI, foot–ground impact; VL, Vastus Lateralis muscle; EMG, electromyographic results; ACC, acceleration; TMV, total magnitude of vibration; ST, settling time; MDF, median frequency.

Values are expressed in mean ± standard deviation, % of variation (%*Δ*), and testing of differences between conditions. Significant effect: *p*-value < 0.05 are shown in bold.

Considering the compression effect, the SnPM analysis depicted no difference in FGI between CONT and COMP (Figure 3D). Compression nevertheless reduced the STV amplitude of the *Vastus Lateralis* with a significant effect over the high frequencies of vibration (>50 Hz) (Figure 3B). The total magnitude of vibration and the median frequency of the compressed leg were significantly lower than that of control (*P* = 0.013 and *P* = 0.001, respectively) (Table 2).

No compression or time effect was detected for the peak acceleration or the settling time.

### 3.2 Muscle activation during the exhaustive downhill run

Muscular activation analysis was conducted on 12 participants due to data loss. Just like the accelerometers, it was caused by the high number and intensity of impacts and vibrations, as well as sweat, which led to movement or loss of contact between the EMG sensors and the muscles during the exhaustive downhill run. The mean level of activation during the exhaustive downhill run reached 29.6% of maximal EMG. No time–compression interaction, compression effect, nor time effect were identified (Table 2).

### 3.3 Neuromuscular testing

The neuromuscular testing was carried out on all 20 participants. A statistical analysis of initial conditions did not identify any difference at baseline.

Linear-mixed models showed no time–compression interaction. The main effects are represented in Table 3. No compression effect was detected, but a significant time effect was found for all the neuromuscular variables immediately after the downhill run (POST) (*p*-value <0.001 for all of them). The exhaustive downhill run indeed resulted in a large decrease in MVC torque, as well as %VA, Db100, and Db10 evoked torques and Db10/Db100. After a delay of 2 days (POST-48), significant losses of MVC torque and Db100 evoked torque were still observed, while no difference in other neuromuscular parameters were identified when compared with PRE.

**Table 3:** Magnitude of compression effect and time effect on neuromuscular variables of knee extensors.

Neuro-muscular variables	Compression effect				Time effect						
	CONT	COMP	ES	*p*-value	PRE	POST	POST-48	ES PRE-POST	*p*-value PRE-POST	ES PRE-POST-48	*p*-value PRE-POST-48
MVC (Nm)	320.8 ± 72.0	326.7 ± 80.4	0.075	0.674	370.7 ± 65.5	274.8 ± 61.6	325.7 ± 69.7	−1.47	**<0.001**	−0.651	**0.001**
VA (%)	84.0 ± 14.6	85.3 ± 12.5	0.094	0.437	91.4 ± 5.7	75.3 ± 15.7	87.3 ± 11.7	−0.95	**<0.001**	−0.373	0.125
Db100 (Nm)	117.8 ± 26.7	120.7 ± 31.5	0.095	0.547	136.9 ± 25.2	95.2 ± 22.6	125.8 ± 22.1	−1.68	**<0.001**	−0.444	**0.005**
Db10 (Nm)	105.3 ± 41.1	109.4 ± 45.6	0.090	0.364	135.7 ± 26.9	58.3 ± 22.0	128.0 ± 27.7	−3.02	**<0.001**	−0.276	0.450
Db10/Db100	0.87 ± 0.22	0.88 ± 0.23	0.042	0.544	0.99 ± 0.09	0.60 ± 0.15	1.03 ± 0.09	−2.88	**<0.001**	0.374	0.088

MVC, maximal voluntary contraction torque; VA, voluntary activation; Db100, 100 Hz doublet evoked torque; Db10, 10 Hz doublet evoked torque.

Values are expressed in mean ± standard deviation, ES, and testing of differences between conditions. Significant effect: *p*-value < 0.05 are shown in bold.

**Figure 4:**
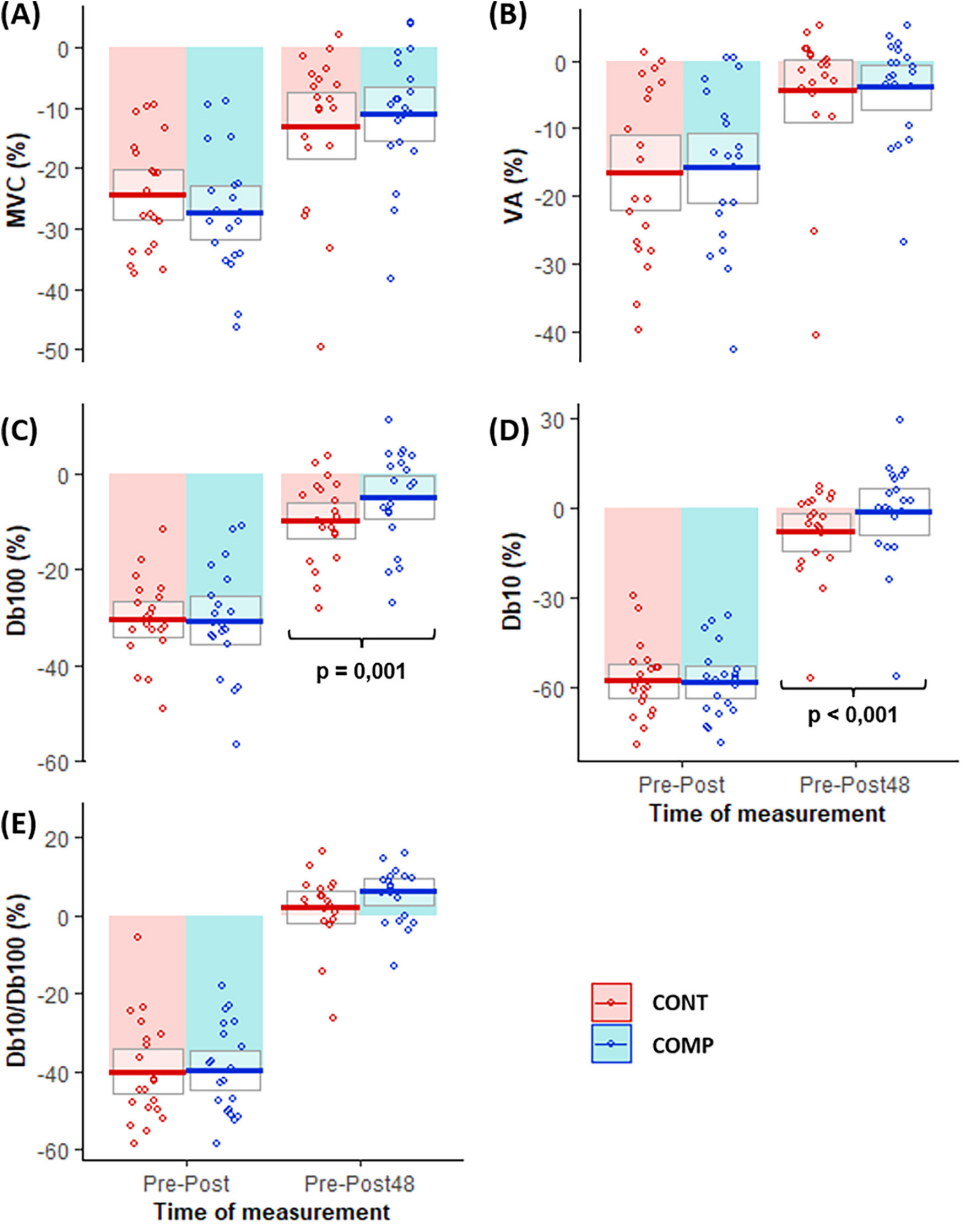
Neuromuscular function changes (%) over time from PRE to POST and PRE to POST-48 in compressed (COMP in blue) and control (CONT in red) conditions. The boxes represent mean ± standard deviation and the small circles represent individual behaviors for **(A)** MVC torque, **(B)** %*VA*, **(C)** Db100 evoked torque, **(D)** Db10 evoked torque, and **(E)** Db10/Db100. *P*-value indicated when the difference between COMP and CONT was significant (<0.05).

Considering the percentage of variations of neuromuscular parameters over time (Figure 4), repeated measures *t*-tests depicted that POST-48 Db100 evoked torque was significantly less decreased in COMP than CONT (−5.0 ± 10.0% and −9.8 ± 8.7%, respectively, ES = −0.475, *P* = 0.001) (Figure 4C). A non-parametric Wilcoxon test revealed a significantly lower decline of POST-48 Db10 evoked torque in the compression than the control condition (COMP = −0.9 ± 17.5%, CONT = −7.9 ± 14.8%, ES = −0.401, *P* < 0.001) (Figure 4D). With the exception of these observations, no significant differences were identified.

### 3.4 Perceived muscle soreness and fatigue

**Figure 5:**
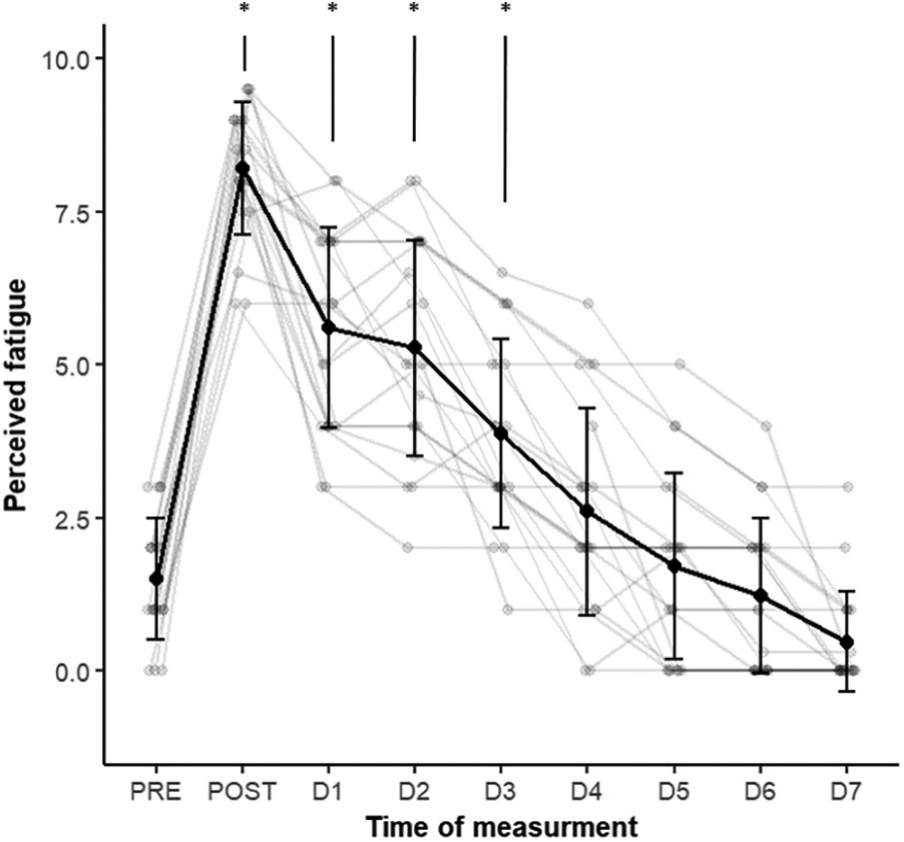
Evolution of perceived fatigue from PRE to the 7th day (D7) after the downhill run. Bold black: trajectory of group mean values ± standard deviation. Thin gray: individual participants’ trajectories. *p < 0.05: significantly different from PRE.

All participants (*n* = 20) completed daily assessments for one week, with the exception of the squat condition of perceived muscle soreness where only 10 provided a full report. The mean value of perceived fatigue reached 8.1 ± 1.1 out of 10 immediately after the downhill run (POST), which was significantly different from PRE = 1.4 ± 1/10 (ES = 4.66, *P* = 0.003) (Figure 5). The increase in perceived fatigue remained elevated until the third day (1.27 < ES < 2.15, *P* < 0.005). By the fourth day, perceived fatigue was no longer different from PRE (Figure 5).

**Figure 6:**
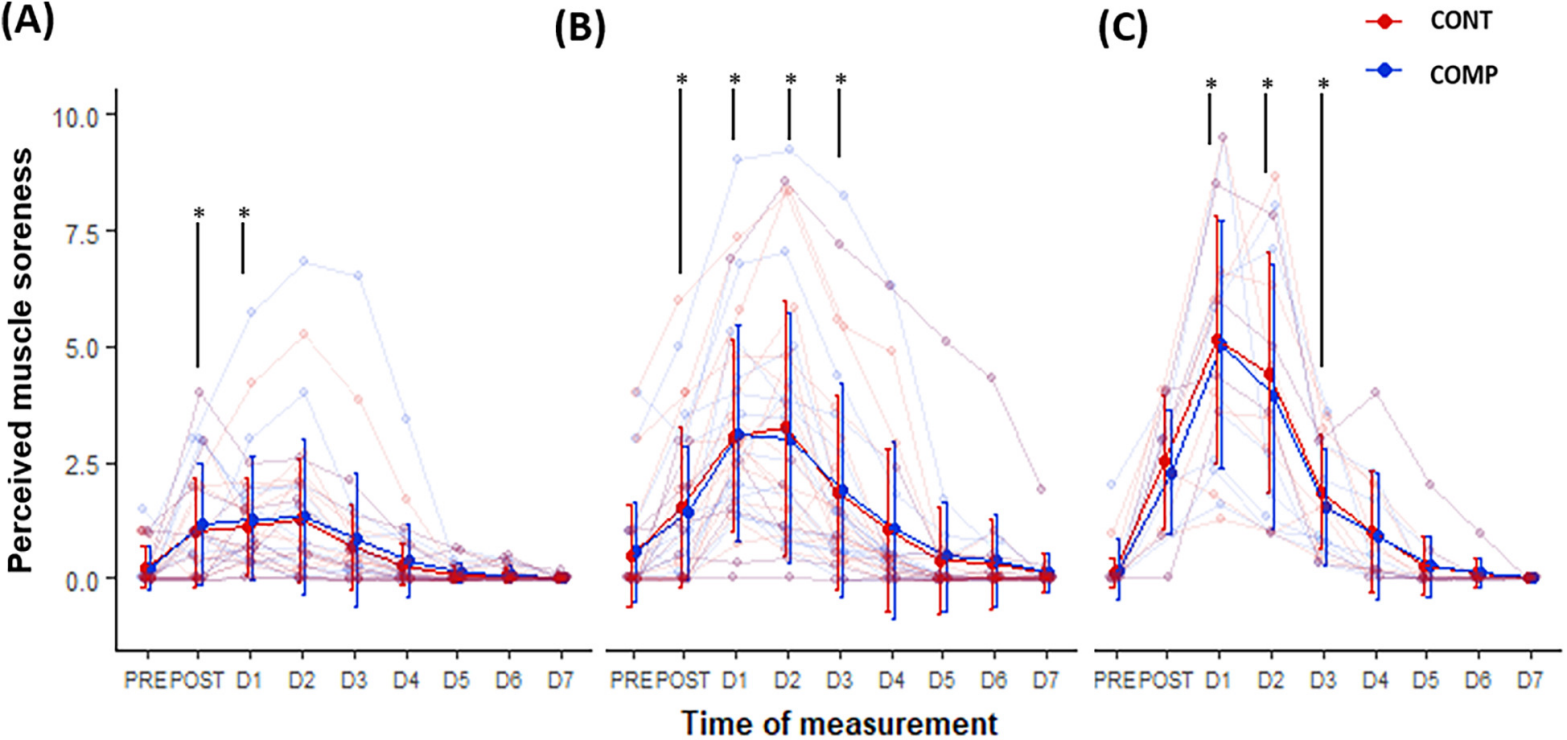
Evolution of perceived muscle soreness from PRE to the 7th day (D7) after the downhill run in compressed (COMP in blue) and control (CONT in red) conditions. From left to right **(A)** in seated **(B)** stretched and **(C)** squat condition. Bold lines: trajectory of group mean values ± standard deviation. Thin lines: individual participants’ trajectories. *p < 0.05: significantly different from PRE.

No time–compression interaction nor compression effect was observed for perceived muscle soreness. A time effect was observed in seated condition (Figure 6A), which rose significantly compared with baseline at day 1 (ES = 0.95, *P* = 0.006) and day 2 (ES = 0.837, *P* = 0.002). When stretching the quadriceps (Figure 6B), perceived muscle soreness on the *Vastus Lateralis* was elevated compared with PRE (0.5 ± 1.1) immediately after downhill run and until the third day (0.568 < ES < 1.21, *P* < 0.02). The differences compared with baseline during the squat condition (Figure 6C) also remained statistically significant until the third day (1.4 < ES < 1.92, *P* < 0.05).

## 4 Discussion

The purpose of this study was to investigate whether wearing high-pressure compression garments on the thigh during downhill running minimizes soft tissue vibrations and, consequently, acute and/or delayed neuromuscular function alterations. The main results indicated that wearing compression garments induced lower (i) magnitude and frequency of soft tissue vibrations of the *Vastus Lateralis* and (ii) delayed impairments of knee extensors contractility (Db100 and Db10 evoked torques).

### 4.1 Soft tissue vibrations

Prolonged downhill run induced a significant increase in FGI magnitude (17.8%) and median frequency (8.6%), as well as STV magnitude of the *Vastus Lateralis* (19.7%) (Table 2), which aligns with previously observed increases of 10%–30% during level running (2, 31, 38), downhill run (19), and the predictions of the Nikooyan and Zadpoor model (22).

Wearing CG did not influence FGI, but as hypothesized, reduced STV of the *Vastus Lateralis* throughout the exhaustive downhill run. This is in line with earlier studies examining both flat and downhill running (13, 19, 39, 40). To the best of our knowledge, we provided the first time–frequency analysis of STV [from Enders et al. (20) and Trama et al. (25)] under CG. This approach made it possible to establish that the significant drop in the total magnitude of vibration of the *Vastus Lateralis* while wearing CG was not linked to a shorter settling time, but rather to a significant reduction in vibration magnitude at high frequencies (Figure 3), thus decreasing the median frequency (Table 2). With regard to previous studies, this could have been explained by lower muscle activation commonly reported under CG (13). However, no change in muscle activation has been observed in the present study, suggesting a mechanical action of CG on soft tissue viscoelastic behavior.

Considering the mass-spring-damper model used to represent muscle viscoelastic behavior (21), applying compressive forces to this model theoretically increases both its stiffness and viscosity (41). Increased stiffness would lead to higher vibration frequencies, whereas greater viscosity would reduce them (42). As the experimental results of the present study showed a decrease in median frequency of vibration with compression, we hypothesized that the primary effect of CG was increasing the viscosity, therefore acting as a low-pass filter (43).

### 4.2 Fatigue and acute neuromuscular impairments

Participants reported high levels of perceived fatigue (8.2 ± 1.1 out of 10) immediately following the downhill run session, comparable to post-ultra-trail levels (∼7 out of 10) (7). As anticipated, the exhaustive downhill run caused a significant decline in all neuromuscular testing parameters, confirming the onset of muscle fatigue in line with previous findings (19, 27, 30). This tends to confirm the plurifactorial etiology of muscle fatigue, as proposed by Millet et al., from intracellular metabolite accumulation (ADP, P_i_, and H^+^ influencing Db100 and MVC torques) to ionic disturbances (Ca^2+^ and K^+^ influencing Db10 torque and Db10/Db100), and voluntary activation inhibitions through metabosensitive fibers (7).

Consistent with a previous review from Weakley et al., which concluded that the effects of compression on acute neuromuscular alteration were uncertain (48), no difference was observed between the two conditions. Despite numerous studies examining the role of CG on immediate neuromuscular alterations, only two focused on downhill running. Bieuzen et al. reported a smaller decrease in MVC torque under CG (16) and Ehrström et al. observed lower %VA deterioration (19), although without changes in other MVC or contractility parameters (Db100, Db10 evoked torques, and Db10/Db100). The variability in acute neuromuscular impairments between the studies can be attributed to the protocol, the level of trail running experience, and more specifically, the training volume in downhill running (23, 44). Thus, the effect of wearing CG on acute neuromuscular alterations following downhill running requires further investigation.

### 4.3 Delayed neuromuscular impairments

The findings of this study revealed significant and lasting neuromuscular alterations up to 48 h post running. The prolonged strength loss and increased perceived muscle soreness have been mainly attributed to muscle damage (45). Previous studies have linked MVC torque deficits following eccentric exercise to structural damage in muscle fibers (e.g., sarcomere, bearing proteins such as dystrophin or intermediate filament desmin, rupture and dissolution of Z-structures) and impaired excitation–contraction coupling (e.g., damage to T-tubules, sarcoplasmic reticulum and sarcolemma, altered Ca^2+^ release from sarcoplasmic reticulum, and altered myofibrillar sensitivity to Ca^2+^) (9, 46).

In line with previous literature that reported a ∼9% deficit in MVC torque remaining at 48 h (30), participants in this study still exhibited an 11.9% reduction in MVC torque compared with baseline. While other studies have reported only a deficit in MVC (23, 30), our study also showed incomplete recovery of Db100 evoked torque, suggesting that these delayed alterations were likely related to structural alterations of the muscle. This can be confirmed by the significant increase in perceived muscle soreness compared with baseline at 24 and 48 h (in every condition), indicating the onset of an inflammatory syndrome triggering metabo/mechanosensitive nociceptors (afferences III and IV) (46).

Notably, wearing CG led to a significant smaller impairment of Db10 evoked torque at 48 h in the compressed condition compared with the control condition, aligning with Ehrström et al. (19), and the results of the present study also showed a protective effect on Db100 evoked torque. These smaller declines in evoked torques at 48 h likely indicate reduced muscle damage in the compressed condition, in accordance with Valle et al. (18). Because this effect was not observed immediately after the exercise, one can assume that intracellular metabolic disturbances after the exhaustive downhill run were so significant that they alone limited torque production, therefore masking the potential protective effect of CG on muscle damage. Furthermore, the delayed onset of hyperalgesia and inflammation, which typically emerge 1–3 days post exercise, suggests that these mechanisms had not yet developed at the time of the initial assessment but may have played a role in the observed differences at 48 h (47).

In contrast to a “likely positive influence” of compression on perceived muscle soreness reported in Weakley et al.'s review (48), no difference was observed in the present study. This could be explained by the protocol design, which involved the compression of only one leg, potentially complicating the ability of the participants to compare soreness between legs, especially at higher pain levels.

### 4.4 Limitations

The main limitation of the study was that the control leg was left uncovered. Some factors such as soft tissues temperature, cutaneous feedback, and placebo effects may have influenced the results. Designing shorts that covered the control leg without applying compression would have provided better control of these variables potentially affecting our outcomes and strengthened the evidence for the role of soft tissue vibrations on neuromuscular impairments. The unilateral compression could also have induced subtle changes in kinematics, which were not observed despite FGI monitoring.

In addition, while the three runners who sustained the effort for an hour could have influenced the results, we verified that their inclusion did not alter the statistical inferences. With regard to the sample size, the number of participants for whom the EMG signal could be analyzed was lower than the number defined in the power calculation. This may explain why, contrary to previous findings, we did not observe any EMG differences between the compressed leg and the control.

Moreover, while delayed neuromuscular impairments are strongly associated with muscle damage as indirect markers, it would have been preferable to conduct histological analyses of muscle biopsies or Magnetic Resonance Imaging for more precise evidence (9, 18). Although perceived pain and fatigue were monitored for 7 days, it would have been preferable to do the same for all neuromuscular markers, which were only measured immediately after and 48 h after the exhaustive downhill run (27).

### 4.5 Perspectives

The results of this study provide valuable insights for practical applications particularly for novice trail runners who are more prone to muscle damage and encounter significant downhill segments. Indeed, our findings suggest that wearing compression garments can offer protection against delayed neuromuscular impairments of knee extensor for this population. This strategy could complement existing approaches used to mitigate vibrations and acute or delayed neuromuscular alterations, such as footwear modification, adaptation of the downhill running technique, or postexercise recovery protocols. However, since no benefit of reducing soft tissue vibrations was previously observed during flat running (10), it suggests that reducing vibrations may limit muscle damage during activities involving high-magnitude impacts and vibrations. This observation prompts further investigation into the optimal dose of vibration exposure and compression levels to determine the most effective strategies for managing training loads and selecting appropriate equipment.

Moreover, the present study highlights the need for more controlled experimental designs to better isolate the effects of vibrations from those of muscle contractions, particularly eccentric ones during movement. Developing protocols that separate these factors could provide a clearer understanding of how shocks and soft tissue vibrations influence muscle damage and athletic performance.

## 5 Conclusion

The present study revealed that wearing high-pressure compression garments reduced delayed alterations in muscle contractile properties after an exhaustive downhill running by acting as a mechanical support for the muscles that attenuated the high frequencies of soft tissue vibrations.

## Data Availability

The raw data supporting the conclusions of this article will be made available by the authors without undue reservation.
